# Long non-coding RNA in glioma: novel genetic players in temozolomide resistance

**DOI:** 10.1080/19768354.2023.2175497

**Published:** 2023-02-15

**Authors:** Jungwook Roh, Mijung Im, JiHoon Kang, BuHyun Youn, Wanyeon Kim

**Affiliations:** a Department of Science Education, Korea National University of Education, Cheongju-si, Republic of Korea; b Department of Hematology and Medical Oncology, Winship Cancer Institute of Emory, Emory University School of Medicine, Atlanta, GA, USA; c Department of Biological Sciences, Pusan National University, Busan, Republic of Korea; d Department of Biology Education, Korea National University of Education, Cheongju-si, Republic of Korea

**Keywords:** Isocitrate dehydrogenase, long non-coding RNA, glioma, temozolomide resistance

## Abstract

Glioma is the most common primary malignant brain tumor in adults and accounts for approximately 80% of brain and central nervous system tumors. In 2021, the World Health Organization (WHO) published a new taxonomy for glioma based on its histological features and molecular alterations. Isocitrate dehydrogenase (IDH) catalyzes the decarboxylation of isocitrate, a critical metabolic reaction in energy generation in cells. Mutations in the IDH genes interrupt cell differentiation and serve as molecular biomarkers that can be used to classify gliomas. For example, the mutant IDH is widely detected in low-grade gliomas, whereas the wild type is in high-grade ones, including glioblastomas. Long non-coding RNAs (lncRNAs) are epigenetically involved in gene expression and contribute to glioma development. To investigate the potential use of lncRNAs as biomarkers, we examined lncRNA dysregulation dependent on the IDH mutation status. We found that several lncRNAs, namely, AL606760.2, H19, MALAT1, PVT1 and SBF2-AS1 may function as glioma risk factors, whereas AC068643.1, AC079228.1, DGCR5, FAM13A-AS1, HAR1A and WDFY3-AS2 may have protective effects. Notably, H19, MALAT1, PVT1, and SBF2-AS1 have been associated with temozolomide resistance in glioma patients. This review study suggests that targeting glioma-associated lncRNAs might aid the treatment of glioma.

## Introduction

Gliomas are primary brain tumors derived from neuroglial stem cells or neuroglial progenitor cells (Weller et al. 2015). The grading of gliomas based on histological features has been debated for decades by technical limitations and different opinions. However, recent advances in molecular genetics have revealed novel changes in several tumors and have changed the diagnostic paradigm for tumors of the central nervous system (CNS). Molecular diagnosis was first introduced in the World Health Organization (WHO) CNS glioma classification system published in 2016 (Louis et al. 2016; Louis et al. 2021). According to this system, grades 1 and 2 are low-glioma grades (LGGs), and grades 3 and 4 are high-glioma grades (HGGs) (Chen et al. 2022). In addition, a new classification in the WHO published in 2021 incorporates more molecular alterations into the diagnosis of many tumors and reclassifies gliomas into adult-type diffuse gliomas, pediatric-type diffuse LGGs, pediatric-type diffuse HGGs, circumscribed astrocytic gliomas, and ependymal tumors (Horbinski et al. 2022). This system also classifies gliomas according to isocitrate dehydrogenase (IDH) mutation status and uses various molecular markers to classify subgroups by their molecular characteristics (Komori 2022).

IDH is an essential enzyme involved in major metabolic processes such as the TCA cycle, glutamine metabolism, adipogenesis, and redox regulation and has three isoforms, and IDH1 and IDH2 are used as glioma biomarkers (Koh et al. 2004; Lee et al. 2004; Yan et al. 2009). The most common mutation detected in human IDH1 replaces arginine at position 132 by histidine (R132H), whereas the one in human IDH2 does arginine at 172 by lysine (R172 K) (Dang et al. 2009; Yan et al. 2009). Wildtype IDH enzymes convert isocitrate to α-ketoglutarate (α-KG). However, the mutant IDHs have changed their substrate specificity, converting α-KG to 2-hydroxyglutarate (2-HG). The consequent excessive accumulation of 2-HG leads to glioma development and their malignant progression, associated metabolic alterations, DNA methylation, and reactive oxygen species (ROS) production (Dang et al. 2009; Lu et al. 2012; Turcan et al. 2012). The majority of LGG patients have mutations in IDH, and a significant correlation has been reported between IDH mutation and improved overall survival in LGG (Rickert 1987; Youssef and Miller 2020). WHO CNS grade 4 glioblastoma can be classified as primary or secondary. Most primary and secondary glioblastomas are of the IDH-wildtype or mutant types, respectively, and overall survival is better for the secondary type (Nobusawa et al. 2009; Wick et al. 2009; Sun et al. 2013). However, diagnoses of glioma based on the mutation status of the IDH genes alone overlook the various origins and characteristics of glioma. Therefore, an understanding of the molecular mechanism of glioma is required for accurate diagnosis and treatment.

Long non-coding RNAs (lncRNAs), which do not encode proteins and are more than 200 nucleotides in length, regulate cellular biological processes in cancer such as cell proliferation, metastasis, invasion, motility, and drug resistance, and their intracellular locations reflect their functions (Seo et al. 2019; Seo et al. 2021; Chen et al. 2022). Nuclear lncRNAs affect gene expression at the transcriptional level, regulating histone modification, DNA methylation and chromatin remodeling (Marín-Béjar et al. 2013; Zhang et al. 2014; Chu et al. 2015; Do et al. 2019). On the other hand, cytoplasmic lncRNAs are competitive endogenous RNAs (ceRNAs) that interact with mRNAs and miRNAs to epigenetically regulate gene expression at the post-transcriptional and translational levels. Recent studies suggest that dysregulations of lncRNA can result in the initiation, progression, and other malignant phenotypes of glioma (Karreth et al. 2011; Tay et al. 2014; Shao et al. 2016; Peng et al. 2018; Zhou et al. 2020).

O^6^-methylguanine-DNA methyltransferase (MGMT) methylation, 1p/19q co-deletion, IDH, and epidermal growth factor receptor have been widely used as glioma biomarkers (McNamara et al. 2013; Ludwig and Kornblum 2017). Recently, it has been demonstrated that lncRNAs might also function as molecular biomarkers in glioma (Peng et al. 2018). For example, lncRNA dysregulation has been shown to be correlated with glioma malignancy and associated with resistance to radiotherapy and temozolomide (TMZ) (Lu et al. 2020; Chae et al. 2021; Kuang et al. 2021; Li et al. 2021). In this review, to address the issue of TMZ resistance, we summarize the effects of lncRNA dysregulation according to IDH mutation status in glioma.

## IDHs as classification biomarkers in glioma

IDH is a representative biomarker of glioma classification, and WHO primarily classifies gliomas according to IDH mutation status. IDH is an enzyme that catalyzes the reversible oxidative decarboxylation of isocitrate, which results in the production of carbon dioxide and α-KG in the TCA cycle during glucose metabolism, which in turn, is coupled with the transformation of NAD(P)^+^ to NAD(P)H (Zhao et al. 2009). In mammals, IDH exists in three isoforms: IDH1 is present in the cytoplasm and peroxisomes, and IDH2 and IDH3, in the mitochondrial matrix (Leighton et al. 1969). IDH1 and IDH2 use NADP^+^ as a cofactor to produce α-KG and NADPH, and the generated NADPH protects cells from oxidative damage by participating in key ROS scavenging processes such as biosynthetic reactions and glutathione disulfide reduction (Hurley et al. 1991; Xu et al. 2004; Itsumi et al. 2015). IDH3 plays a central role in energy production in the TCA cycle by generating α-KG and NADH using NAD^ +^ as a cofactor (Reitman and Yan 2010). Both IDH1 and IDH2 form a homodimer, whereas IDH3 holoenzyme is multimeric and assembled with multiple subunits of α, β, and γ encoded by IDH3a, IDH3b, and IDH3c, respectively (MacDonald et al. 2013). In diffuse gliomas, the IDH1-mutant type is the most common and observed in more than 90% of cases. The IDH2-mutant type is observed in approximately 10% of cases, but the IDH3-mutant type has never been observed (Waitkus et al. 2018).

In cases of IDH1 mutation, the homodimer formed by two IDH1-mutant monomers does not have enzymatic activity, but the heterodimer composed of the IDH-wildtype and mutant monomer has enzymatic activity sufficient to convert α-KG to 2-HG (Dang et al. 2009; Yang et al. 2010). IDH is one of the most studied genes in glioma development, and cancers with IDH1 or IDH2 mutations produce 10–100 times more 2-HG than those with the IDH1 or IDH2 wildtype (Ward et al. 2010; Leeper et al. 2015). IDH1 mutation converts α-KG to 2-HG, which accumulates in the cytoplasm, and thereby releases carbon from the TCA cycle. This cycle utilizes metabolic reprogramming to compensate for fluctuations in metabolic pathways; for example, glutamate dehydrogenase converts glutamine to α-KG, which replenishes the TCA cycle (Hausinger 2004; Loenarz and Schofield 2008; Xu et al. 2011). In addition to the metabolic changes, the hypermethylation of CpG islands is also affected by IDH mutations. The glioma-specific methylation pattern provides clues regarding the pathogenesis of IDH-mutant gliomas. DNA methylation is regulated by methyltransferase and demethylase. During demethylation, ten-eleven translocation methylcytosine dioxygenase (TET) converts 5-methylcytosine to cytosine in an iron- and α-KG-dependent manner. Furthermore, the activity of TET might be inhibited by 2-HG due to its structural similarity to α-KG (Xu et al. 2011; Lee et al. 2021). Histone methylation status is regulated by histone methyltransferases such as euchromatic histone lysine methyltransferase 1 (EHMT1), EHMT2, SET nuclear proto-oncogene (SET), and enhancer of zeste 2 polycomb repressive complex 2 subunit (EZH2), and demethylases such as lysine demethylase 1A (KDM1A) and KDM4A (Chowdhury et al. 2011; Hayward and Cole 2016; Katoh 2016; Rahman et al. 2021). Like TET, histone demethylases such as KDM4 and KDM5 are inhibited by high levels of 2-HG (Chowdhury et al. 2011). Many studies report that histone methylation markers accumulate in various cancers harboring a mutant IDH gene. Also, inhibition of histone demethylation by 2-HG leads to impaired cell differentiation, which might be associated with the tumorigenesis of IDH-mutant glioma (Lu et al. 2012).

IDH is an essential enzyme for energy metabolism associated with the TCA cycle, and mutated IDH causes 2-HG accumulation, hypermethylation, and oxidative damage. Because of these alterations, it is reasonable to classify glioblastomas by IDH mutational status. The new molecular markers including IDH will help diagnose and effectively treat gliomas.

## LncRNAs and IDH mutations in glioma

In brain tumors, IDH mutation status is known to be associated with a patient’s prognosis of brain tumors and with a well-known molecular biomarker of glioma (Turcan et al. 2012). Reportedly, lncRNAs are epigenetically involved in gene expression regulation and treatment resistance in brain tumors (Mercer et al. 2009). Furthermore, accumulating evidence suggests that lncRNAs function as ceRNAs that regulate gene expression and alter cell biological properties such as cell viability, proliferation, motility, and invasion (Do and Kim 2018; Seo et al. 2020).

## Dysfunction of lncRNAs according to IDH-mutant status

Glioblastoma can be classified as IDH-wildtype or mutant types, each of which is associated with a distinct tumor behavior and prognosis. LncRNA AC068643.1 is more highly expressed in IDH-mutant glioblastomas than in the wildtype. The expression levels of AC068643.1 are positively correlated with the protein levels of bone morphogenetic protein (BMP) and myostatin (MSTN), and it has been experimentally demonstrated that both BMP and MSTN directly stimulate AC068643.1 expression (Huang et al. 2020). BMP inhibits cell proliferation in glioblastoma and astrocytic glioma, and a BMP-like synthetic is considered a non-cytotoxic therapeutic that might prevent glioma growth and recurrence. Altogether, these results suggest that AC068643.1 can function as a protective lncRNA in glioblastoma patients (González-Gómez et al. 2014; Sachdeva et al. 2019).

Differentially expressed lncRNAs were identified using GSE107850, a dataset of glioma patients that underwent surgical excision and TMZ treatment. A risky lncRNA AL606760.2 and two protective lncRNAs, FAM13A antisense RNA 1 (FAM13A-AS1) and AC079228.1, were suggested as predictors of TMZ efficacy in IDH-mutant type LGGs (Li et al. 2021). Furthermore, levels of AL606760.2 and Smad2, which mediates transforming growth factor (TGF)-beta signaling, are positively correlated. Thus, AL606760.2 appears to regulate intracellular processes such as cell proliferation, apoptosis, and differentiation (Abdel-Wahab et al. 2002). On the other hand, FAM13A-AS1 is involved in decreasing cell proliferation and differentiation, and its levels were found to be significantly correlated with UBR5 levels (an E3 ubiquitin-protein ligase) (Jiang et al. 2011; Gudjonsson et al. 2012). The two cancer-related genes, pre-mRNA processing factor 40 homolog B (PRPF40B) and zinc finger homeobox 3 (ZFHX3), were suggested to be potential target mRNAs of lncRNA AC079228.1. PRPF40B is a splicing gene that directly interacts with splicing factor 1 (SF1), and associated with U2 small nuclear RNA auxiliary factor 1 (U2AF1). ZFHX3 acts as a transcription factor for regulation of myogenic and neuronal differentiation and inhibition of cell cycle progression (Benjamin et al. 2009; Gudbjartsson et al. 2009).

In another study, lncRNAs differentially expressed in IDH-mutant and IDH-wildtype glioma samples obtained from several CCGA, TCGA, and GSE16011 datasets were analyzed using the LNCipedia database (Chen et al. 2020). Multivariate Cox regression analysis of the prognostic performances of differentially expressed lncRNAs identified H19 and plasmacytoma variant translocation 1 (PVT1) as risky lncRNAs and highly accelerated region 1A (HAR1A) as a protective lncRNA in glioma. LncRNA H19 promotes angiogenesis in gliomas by increasing the expressions of hypoxia inducible factor 1 subunit alpha (HIF-1α) and vascular endothelial growth factor (VEGF) through targeting miR-138. In glioma, H19 can also target miR-342 and upregulate the Wnt5a/β-catenin pathway to promote cell proliferation and migration (Liu et al. 2020; Zhou et al. 2022). Also, lncRNA PVT1 targets miR-200a to regulate the cell cycle and promote cell proliferation and invasion (Zhang et al. 2019). On the other hand, lncRNA HAR1A acts to suppress the progression of diffuse glioma and is expressed at low levels in glioma. In addition, glioma patients with high PVT1 expression or low HAR1A expression is reported to be prognostic of poor survival in glioma patients. Moreover, the down-regulation of PVT1 and up-regulation of HAR1A improved the survival of glioma patients that received chemotherapy and radiotherapy. These findings imply that these lncRNAs play critical roles in diffuse glioma progression and that PVT1 and HAR1A should be explored as promising biomarkers for diagnosis, prognosis, and targeted therapy (Zou et al. 2017).

## LncRNA dysfunction in IDH-wildtype gliomas

IDH-wildtype gliomas are associated with poor prognoses and high CNS grades. Metastasis-associated lung adenocarcinoma transcript 1 (MALAT1) is an overexpressed lncRNA in glioblastoma and promotes cell growth and tumorigenesis. For example, it has been demonstrated that lncRNA MALAT1 promotes the expression of SRY-box transcription factor 2 (SOX2) by suppressing miR-129 to promote glioma stem cell viability, proliferative abilities, and tumorigenesis (Xiong et al. 2018). In addition, methyltransferase 3 (METTL3), which upregulates the expression of MALAT1 through N^6^-methyladenosine (m^6^A) modification, is also associated with poor prognosis. It was also demonstrated that MALAT1 overexpression promotes the malignant glioma phenotype by inducing epithelial–mesenchymal transition (EMT) and tumor necrosis factor (TNF) signaling through nuclear factor kappa B (NF-κB) activation (Chang et al. 2021).

Furthermore, lncRNA SBF2 antisense RNA 1 (SBF2-AS1), which plays an oncogenic role in various tumors, may function as a biomarker in diffuse LGG. Kaplan-Meier analysis showed that the prognosis of LGG patients with high SBF2-AS1 expression was poorer than those with low expression. Cox regression analysis showed that SBF2-AS1 is an independent prognostic factor of poorer overall survival in LGG (Zhang et al. 2021). Most LGGs are IDH mutant type, and only some are IDH-wildtype. However, the molecular and clinical characteristics of IDH-wildtype LGG are similar to those of glioblastoma. It is reported that SBF2-AS1 is more highly expressed in IDH-wildtype than in IDH-mutant glioma and its knockdown in glioblastoma upregulates miR-338-3p, but downregulates EGF-like domain multiple 7 (EGFL7), thereby inhibiting angiogenesis (Cancer Genome Atlas Research Network et al. 2015; Yu et al. 2017; Zhang et al. 2021).

An analysis of the CGCA database revealed lncRNA WDFY3 antisense RNA 2 (WDFY3-AS2) is expressed at low levels in IDH-wildtype gliomas. Kaplan-Meier analysis showed that WDFY3-AS2 overexpression was associated with longer overall survival than low WDFY3-AS2 expression, and Cox regression analysis showed its expression was independently correlated with overall survival. In addition, gene ontology (GO) and gene set enrichment analysis (GSEA) revealed that WDFY3-AS2 is involved in the regulation of synaptic transduction, glutamate receptors, and TNF signaling pathways (Wu et al. 2018).

LncRNA DiGeorge syndrome critical region gene 5 (DGCR5) plays cancer-dependent roles. For example, although DGCR5 levels were unrelated to WHO malignancy grade in glioma, they were more downregulated in IDH-wildtype glioma than in IDH-mutant glioma. Furthermore, DGCR5 increased the levels of Smad7 and phosphatase and tensin homolog (PTEN) by sponging miR-21 and miR-23a, respectively, in glioma cells. It is also reported that Smad7 inhibits the migratory and invasive activities of glioma, and PTEN inhibits cell proliferation and enhances apoptosis. Patients with low DGCR5 expression levels show poorer overall survival (He et al. 2020).

## TMZ resistance by lncRNAs according to IDH mutation status

Glioma classification based on histological features and IDH mutation status helps with diagnosis and prognosis. Although advances in techniques, technologies, surgical resection, chemotherapy, and radiotherapy have significantly improved treatment efficacy outcomes fall far short of satisfactory. Furthermore, accumulating evidence indicates lncRNA dysregulations are involved in glioma chemo- and radioresistance. This review compiles several differentially expressed lncRNAs in IDH-mutant and IDH-wildtype gliomas, and it has been shown that lncRNAs SBF2-AS1, PVT1, MALAT1, and H19 significantly contribute to the induction of TMZ resistance.

TMZ is a lipophilic anticancer drug, which is widely used to treat brain tumors. TMZ can be readily delivered to brain tissues as it crosses the blood–brain barrier and induces cell death by causing single- or double-strand breaks by incorporating mismatched base pairs. However, more than 50% of glioma patients do not respond to TMZ, and lncRNAs appear to be involved in TMZ resistance by regulating chemoresistance-associated gene expression, enhancing cell survival, and inhibiting apoptosis (Lee 2016). Furthermore, numerous studies have suggested that lncRNA might act as a ceRNA of miRNA to modulate the expressions of target genes (Salmena et al. 2011). The interaction between lncRNA and miRNA might also influence TMZ resistance in glioma cells. It is reported that sponging of miR-151a-3p by lncRNA SBF2-AS1, which is more highly expressed in IDH-wildtype than IDH-mutant gliomas, is responsible for TMZ resistance in glioma cells (Zhang et al. 2019). Furthermore the transcription factor, zinc finger E-box binding homeobox 1 (ZEB1), might increase SBF2-AS1 expression by binding to its promotor region, and the resulting high level of SBF2-AS1 downregulates miR-151a-3p and the subsequent upregulation of X-ray repair cross complementing 4 (XRCC4), a target of miR-151a-3p. XRCC4 promotes DNA double-strand break repair and thus enhances cell survival and inhibits apoptosis in glioma cells in response to TMZ treatment. Furthermore, it has been shown that SBF2-AS1 depletion reduces the level of XRCC4, delays the repair of TMZ-induced DNA damage, and increases glioma cell sensitivity to TMZ. PVT1 is a known oncogene in various cancers, including glioma. It is expressed at higher levels in IDH-mutant than in IDH-wildtype gliomas and reportedly, its knockdown effectively promotes glioma cell sensitivity to TMZ. PVT1 and miR-365 expressions are negatively correlated, and the overexpression of miR-365 suppresses TMZ resistance in glioma cells. PVT1 competitively binds to miR-365 and positively regulates the expression of E74 like ETS transcription factor 4 (ELF4), which contributes to the induction of glioma stemness and TMZ resistance (Bazzoli et al. 2012; Gong et al. 2021). MALAT1 is frequently overexpressed in IDH-wildtype gliomas and enhances resistance to TMZ administration by regulating the expressions of miRNAs. Several studies have shown that miR-203 contributes to reduced chemoresistance in various cancers (Li et al. 2011; Lin et al. 2016; Zhang et al. 2016). Furthermore, MALAT1 promotes glioma cell proliferation and TMZ resistance by decreasing miR-203 expression and increasing the expression of thymidylate synthase (TS), a potent cancer chemotherapy target (Chu et al. 2003; Chen et al. 2017). In addition, MALAT1 downregulates miR-101, consequently increasing resistance to TMZ in glioma cells by increasing glycogen synthase kinase 3β (GSK-3β) levels. Also, increased GSK-3β expression might be associated with resistance to TMZ and poor prognosis in glioma patients by decreasing MGMT promoter methylation and thus upregulation of MGMT (Pyko et al. 2013; Tian et al. 2016; Cai et al. 2018). MALAT1 also regulates TMZ resistance by modulating multidrug resistance-related genes. The downregulation of MALAT1 reduces the mRNA expression levels of multidrug resistance-related proteins such as multidrug resistance protein 1 (MDR1), multidrug resistance protein 5 (MRP5), and lung–resistance related protein 1 (LRP1), which are important regulators of chemoresistance, and also reduces glioma cell sensitivity to TMZ. In addition, MALAT1 is involved in EMT induction and thus regulates glioblastoma cell sensitivity to TMZ. The downregulation of MALAT1 decreases TMZ resistance by reducing the expression level of ZEB1, an EMT-related protein that increases cancer cell motility and invasiveness (de Cremoux et al. 2007; Li et al. 2017; Dong et al. 2019). H19 is highly expressed in IDH-mutant gliomas and involved in TMZ resistance by regulating multidrug resistance genes, and its levels are elevated in TMZ-resistant glioma cells. Inhibition of H19 downregulates the expressions of several multidrug resistance genes such as MDR1, MRP1, and ATP-binding cassette super-family G member 2 (ABCG2) in TMZ-resistant cells at the mRNA and protein levels, which suggests H19 plays an important role in the induction of TMZ resistance in glioma cells (Jiang et al. 2016). Furthermore, oxidative stress induces H19 expression and thus increases cell survival and viability by activating NF-κB signaling, which accompanies the acquisition of TMZ resistance by glioma cells (Duan et al. 2018).

**Figure 1.**
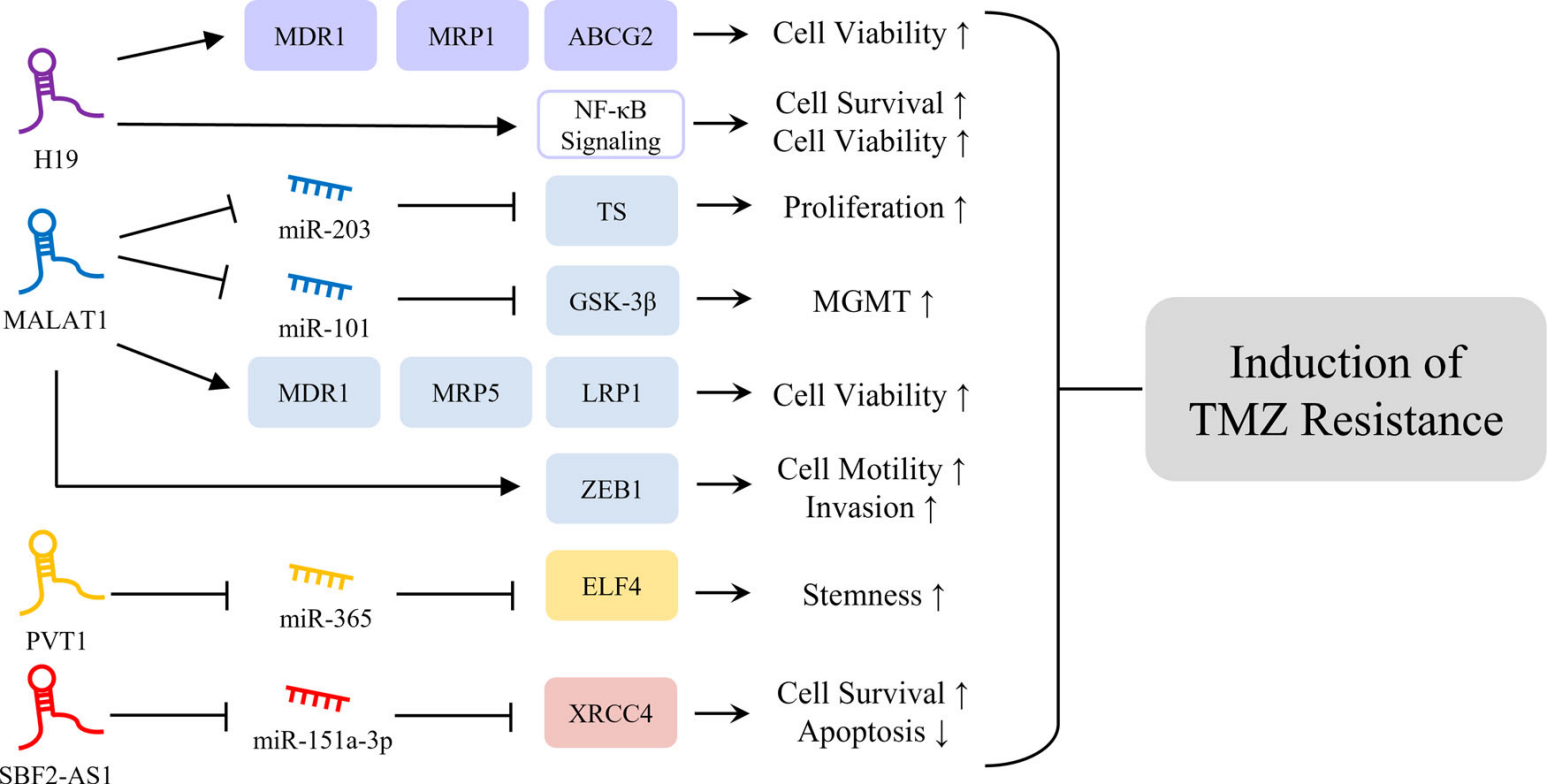
Mechanisms of lncRNAs inducing TMZ resistance. H19 is involved in induction of glioma cell viability and survival through upregulation of MDR1, MRP1, and ABCG2 and activation of NF-κB signaling, responsible for induction of TMZ resistance (Jiang et al. 2016; Duan et al. 2018). MALAT1 is associated with glioma cell proliferation, viability, and invasion through regulation of ceRNA networks with miRNAs, upregulation of multidrug resistance-related genes, and regulation of ZEB1, which might contribute to glioma TMZ resistance (Chu et al. 2003; de Cremoux et al. 2007; Pyko et al. 2013; Tian et al. 2016; Chen et al. 2017; Li et al. 2017; Cai et al. 2018; Dong et al. 2019). PVT1 and SBF2-AS1 play oncogenic roles in induction of glioma stemness and cell survival, respectively, through regulation of ceRNA networks, resulting in TMZ resistance in glioma cells (Bazzoli et al. 2012; Zhang et al. 2019; Gong et al. 2021).

In glioma, increased TMZ resistance, conferred by the upregulations of risky lncRNAs such as H19, MALAT1, PVT1, and SBF2-AS1 (Figure 1), is likely to play a key role in the therapeutic efficacy of surgical resection plus drug treatment. However, the discovery of lncRNAs, known to induce TMZ resistance, does not completely explain the difficulties of treating patients with TMZ-resistant glioma. Nonetheless, targeting these lncRNAs is a promising way of reducing TMZ resistance.

## Conclusions

**Figure 2.**
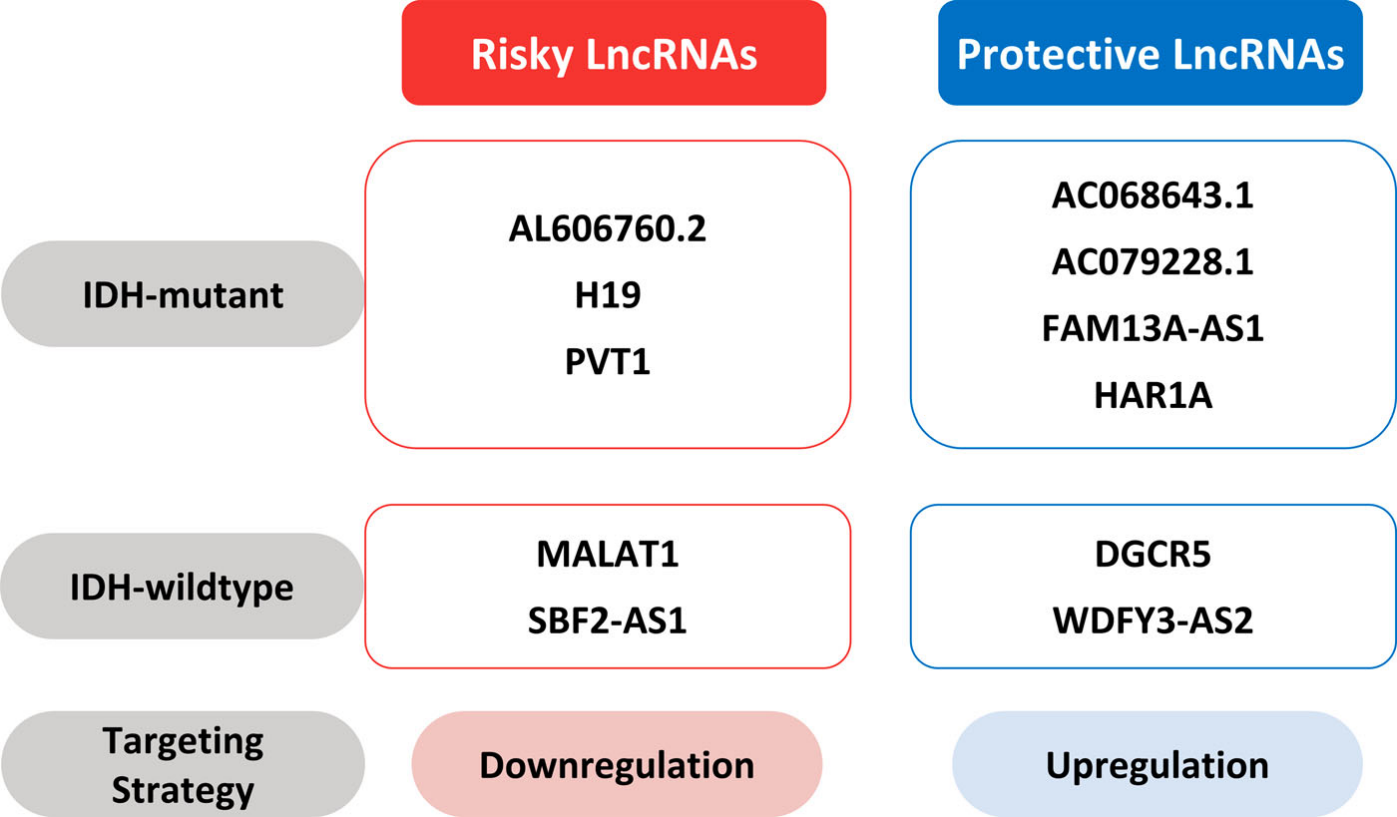
Therapeutic strategies targeting lncRNAs. Downregulation of risky lncRNAs such as AL606760.2 (Li et al. 2021), H19 (Chen et al. 2020), PVT1 (Chen et al. 2020), MALAT1 (Xiong et al. 2018), and SBF2-AS1 (Zhang et al. 2021) and upregulation of protective lncRNAs such as AC068643.1 (Huang et al. 2020), AC079228.1 (Li et al. 2021), FAM13A-AS1 (Li et al. 2021), HAR1A (Chen et al. 2020), DGCR5 (He et al. 2020), and WDFY3-AS2 (Wu et al. 2018) might help predict prognosis in glioma.

The dysregulation of lncRNA contributes to the development of several types of cancer, and lncRNA promotes glioma development and induces chemo- and radioresistance. IDH mutation status is a widely-accepted biomarker for glioma classification, but additional markers are desired to determine effective treatment strategies. This review focuses on risky and protective lncRNAs according to IDH mutation status and the mechanisms involved in TMZ resistance. We suggest five lncRNAs acting as risk factors, namely, AL606760.2, H19, MALAT1, PVT1, and SBF2-AS1, and another six protective lncRNAs, that are AC068643.1, AC079228.1, DGCR5, FAM13A-AS1, HAR1A, and WDFY3-AS2. The risky H19, MALAT1, PVT1, and SBF2-AS1 increase TMZ resistance (Figure 2). Identifying these risky or protective lncRNAs should help predict prognosis in glioma. Determining the dysregulation statuses of lncRNAs associated with TMZ resistance should aid the development of promising pharmacological targets. Therefore, therapeutic strategies targeting the dysregulation of lncRNA according to IDH mutation status will help improve the prognoses of glioma patients.
